# TIFA protein expression is associated with pulmonary arterial hypertension

**DOI:** 10.1038/s41598-021-93582-1

**Published:** 2021-07-08

**Authors:** Hao-Chih Chang, Tong-You Wade Wei, Pei-Yu Wu, Ming-Daw Tsai, Wen-Chung Yu, Chen-Huan Chen, Shih-Hsien Sung

**Affiliations:** 1 Division of Cardiology, Department of Medicine, Taipei Veterans General Hospital, No. 201, Sec. 2, Shipai Road, Beitou District, Taipei, Taiwan; 2 Cardiovascular Research Center, National Yang Ming Chiao Tung University, Taipei, Taiwan; 3 Department of Internal Medicine, National Yang Ming Chiao Tung University College of Medicine, Taipei, Taiwan; 4 Institute of Public Health, National Yang Ming Chiao Tung University College of Medicine, Taipei, Taiwan; 5 Institute of Biological Chemistry, Academia Sinica, Taipei, Taiwan; 6 Deparment of Medicine, Taipei Veterans General Hospital Yuanshan and Suao Branch, Yilan, Taiwan

**Keywords:** Biomarkers, Cardiology, Diseases

## Abstract

Tumor necrosis factor receptor-associated factor-interacting protein with a forkhead-associated domain (TIFA), a key regulator of inflammation, may be involved in the pathogenesis of pulmonary arterial hypertension (PAH). A total of 48 PAH patients (age 50.1 ± 13.1 years, 22.9% men), 25 hypertensive subjects, and 26 healthy controls were enrolled. TIFA protein expression in peripheral blood mononuclear cells (PBMCs) and plasma interleukin (IL)-1β and tumor necrosis factor (TNF)-α were measured. Pulmonary arterial hemodynamics were derived from right heart catheterization. PAH patients had the highest expression of TIFA, TNF-α, and IL-1β. TIFA protein expression was significantly associated with IL-1β (r = 0.94; *P* < 0.001), TNF-α (r = 0.93; *P* < 0.001), mean pulmonary artery pressure (r = 0.41; *P* = 0.006), and pulmonary vascular resistance (r = 0.41; *P* = 0.007). TIFA protein expression could independently predict the presence of PAH (odds ratio [95% confidence interval per-0.1 standard deviation]: 1.72 [1.37–2.16]; *P* < 0.001) and outperformed echocardiographic estimation. Ex vivo silencing of TIFA protein expression in PBMCs led to the suppression of the cellular expression of IL-1β and TNF-α. IL-1β and TNF-α mediated 80.4% and 56.6% of the causal relationship between TIFA and PAH, respectively, supporting the idea that TIFA protein is involved in the pathogenesis of PAH.

## Introduction

Pulmonary arterial hypertension (PAH), classified as group 1 pulmonary hypertension by the World Health Organization (WHO), causes a devastating decline in the cardiac function and is associated with poor prognosis if untreated. Although great advancements have been achieved in early diagnosis and pharmacological therapies in the past two decades, the 5-year survival rate (57%) remains low^1,2^. The pathobiology of PAH involves the remodeling of the pulmonary vasculature that includes vasoconstriction and smooth muscle cell proliferation^3^. Accumulating evidence suggests the pathogenic role of inflammation in mediating deteriorated vascular remodeling in PAH^4,5^. Soon et al*.* demonstrated significantly higher levels of tumor necrosis factor (TNF)-α, interleukin (IL)-1β, and IL-6 in 58 patients with idiopathic PAH than in healthy controls^6,7^. Higher serum levels of TNF-α, IL-1β, and IL-6 were associated with worse pulmonary vascular resistance (PVR) and cardiac index (CI), and IL-6 was predictive of clinical outcomes^6,8–10^. In addition, reduction in inflammatory mediators after treatment indicated improvement in functional class and long-term survival^11^.

Tumor necrosis factor receptor-associated factor (TRAF)-interacting protein with a forkhead-associated (FHA) domain (TIFA), an adapter protein functions as a key signaling transducer and activates nuclear factor kappa B (NF-κB)^12^. Once stimulated by the upstream circulating TNF-α, TIFA is phosphorylated at threonine residual nine and undergoes oligomerization via the intermolecular binding between the FHA domain and the phosphorylated threonine^13^. TIFA oligomerization subsequently activates NF-κB and upregulates the expression of downstream inflammatory cytokines^13^. Although NF-κB upon activation has been reported to initiate the inflammatory cascade in PAH patients^14,15^, whether TIFA protein is involved in the pathogenesis of PAH is yet undetermined. In the present study, we aimed to examine and compare the expression of TIFA in peripheral blood mononuclear cells (PBMCs) from patients with PAH or systemic hypertension, and healthy controls. We also investigated the association between TIFA protein expression, plasma levels of IL-1β and TNF-α, and pulmonary arterial hemodynamics.

## Methods

### Study participants

Patients aged > 18 years who were newly diagnosed with group 1 PAH as per the WHO classification, yet received any PAH-specific medication, were enrolled in this study from August 2016 to June 2018. The diagnosis of PAH was made according to practical guidelines^16^. Patients with either idiopathic PAH or connective tissue disease-associated PAH (CTD-PAH) were further confirmed and included in the final analysis. In addition, subjects referred for the survey of systemic hypertension were enrolled as controls. All the controls were subjected to 24-h blood pressure recording (WatchBP O3, Microlife). Following the criteria of ambulatory blood pressure ≥ 130/80 mmHg of the 24-h average, ≥ 135/85 mmHg of the daytime average, or ≥ 120/70 mmHg of the nighttime average, participants were further classified as hypertensive subjects or healthy controls. This study was approved by the institutional review board at Taipei Veterans General Hospital, Taipei, Taiwan (IRB 2016-01-012AC), and was performed in accordance with relevant guidelines and regulations. Informed consent was obtained from all participants and/or their legal guardians.

### Demography and clinical examinations

A total of 99 participants were analyzed for demographic measures, including body weight and height, blood pressure, and echocardiographic studies. Systolic and diastolic blood pressure (SBP and DBP) were recorded, and mean arterial pressure (MAP) was calculated as the sum of 1/3 SBP + 2/3 DBP. Left ventricular ejection fraction (LVEF), estimated right ventricular systolic pressure (eRVSP), and tricuspid annular plane systolic excursion (TAPSE) were calculated according to the American Society of Echocardiography standard^17^. Fasting blood samples were collected for biochemical analysis, including blood urea nitrogen and creatinine. Estimated glomerular filtration rate (eGFR) was calculated from the Modification of Diet in Renal Disease equation for Asians^18^.

For PAH patients, 6-min walk distance (6MWD) and N-terminal pro-B type natriuretic peptide (NT-proBNP) levels were measured. Right heart catheterization was performed to obtain pulmonary hemodynamics, including pulmonary artery wedge pressure (PAWP), systolic pulmonary artery pressure (sPAP), mean pulmonary artery pressure (mPAP), CI, and PVR.

### Isolation of PBMCs, cell culture, and silencing of TIFA protein expression

A 15-mL whole blood sample was obtained from each participant, and PBMCs and plasma were isolated by density gradient centrifugation using Ficoll-Paque Plus (GE Healthcare, Piscataway, NJ, USA) as previously described^19,20^. PBMCs were then maintained in Roswell Park Memorial Institute (RPMI)-1640 medium (Invitrogen) supplemented with 10% decomplemented fetal bovine serum (FBS; Gibco), 200 mmol/L L-glutamine (Gibco), 100 U/mL penicillin (Gibco), and 100 mg/mL streptomycin (Gibco) in a humidified atmosphere at 37 °C with 5% CO_2_. For small-interfering ribonucleic acid (siRNA)-based gene silencing, a total of 10^6^ PBMCs were transfected with 50 pmol of double-stranded siRNA oligomers corresponding to the sequence 5ʹ-UCA GGA CAA ACA GGU UUC CCG AGU U-3ʹ to target TIFA or control siRNA (synthesized by GenScript) supplemented with 15 µL of Lipofectamine RNAiMAX (Invitrogen) in the presence of Opti-MEM (Invitrogen). After 6 h from primary transfection, cells were incubated in regular medium for 24 h, and secondary siRNA transfection was performed using the same protocol. Cells and the conditioned media were collected after 72 h of incubation.

### Estimations of the TIFA protein expression

To estimate TIFA protein expression in PBMCs, western blot analysis was performed following the previously established method^19,20^. Briefly, patient PBMCs were washed with ice-cold phosphate-buffered saline (PBS), lysed in radioimmunoprecipitation assay (RIPA) lysis buffer (50 mM Tris pH 7.4, 150 mM sodium chloride [NaCl], 1% NP-40, 0.5% sodium deoxycholate, and 0.1% sodium dodecyl sulfate [SDS]) supplemented with a protease inhibitor cocktail (Roche) and phosphatase inhibitor cocktail (Sigma). After five repeated freeze–thaw cycles, the cell extracts were cleared by centrifugation at 4 °C. Cell extracts were quantified using the Bradford assay, and equal amounts of soluble proteins were mixed with SDS sample buffer, boiled, separated by SDS–polyacrylamide gel electrophoresis (PAGE), and blotted onto polyvinylidene fluoride (PVDF) membranes (Millipore). Membranes were then blocked for 1 h with 5% dry milk in PBST (PBS supplemented with 0.1% Tween-20) buffer and incubated in a primary antibody diluted in PBST containing 1% bovine serum albumin (Sigma) for overnight at 4 °C. For detection of TIFA, a homemade anti-TIFA monoclonal antibody was used as previously described^19^. After three washes with PBST, membranes were incubated with a horseradish peroxidase (HRP)-conjugated anti-mouse IgG. Membranes were washed five times with PBST, and the blotted protein bands were revealed using an enhanced chemiluminescence (ECL) system (Millipore). The results of immunoblots were quantified and normalized to corresponding β-actin (Abcam) levels, and relative intensities normalized to normal counterparts were analyzed.

### Measurement of pro-inflammatory cytokines

To assess the levels of cytokines in the plasma or conditioned media of cultured PBMCs, enzyme-linked immunosorbent assay (ELISA) test kits for IL-1β and TNF-α (R&D Systems) were used^19^. The absorbance of triplicate samples was read by SpectraMax Paradigm Plate Reader (Molecular Devices).

### Statistical analysis

The baseline characteristics were reported as mean ± standard deviation for continuous variables and percentages for categorical variables. One-way analysis of variance (ANOVA) was performed to evaluate differences in baseline characteristics between normal subjects, systemic hypertensive patients, and PAH patients. Post-hoc analysis with Bonferroni test was used for between-group comparisons. Linear regression analysis was employed to evaluate the correlation between TIFA protein expression and continuous variables, including IL-1β and TNF-α. A generalized linear model was used to calculate the estimated mean ± standard error of TIFA, IL-1β, and TNF-α in different groups after accounting for age, gender, and MAP. Logistic regression analysis was conducted after standardization and adjusting for age for TIFA, IL-1β, and TNF-α to clarify their role in the prediction of PAH. Receiver operating characteristic (ROC) curve analysis was used to derive the cut-off value of TIFA for the diagnosis of PAH, and the diagnostic powers of TIFA and echocardiographic measures were compared. Mediation analysis was performed to examine the causal–mediation relationship among TIFA, IL-1β, TNF-α, and PAH. Direct and indirect effects and confidence intervals were estimated by bootstrapping with 2,000 resamples. A two-tailed *P* value < 0.05 was considered statistically significant. All statistical analyses were performed using SPSS version 22.0 (IBM, Chicago, IL, USA) and SAS 9.4 (IBM Inc., Cary, NC, USA).

## Results

### Baseline characteristics

A total of 99 subjects were enrolled in this study, including 48 group 1 PAH patients (age 50.1 ± 13.1 years, 22.9% men), 25 hypertensive patients (age 54.1 ± 9.3 years, 68% men), and 26 normal controls (age 57.6 ± 10.4 years, 46.2% men). Among the PAH patients, 32 (66.7%) had idiopathic PAH and 16 (33.3%) had CTD-PAH. The baseline characteristics are shown in Table 1. In brief, the PAH patient group was dominated by women, had lower blood pressure, eGFR, and TAPSE, and higher LVEF and eRVSP, while the subjects with systemic hypertension had higher body mass index and blood pressure.

**Table 1:** Baseline characteristics of the study population.

Characteristic	Normal (n = 26)	Hypertension (n = 25)	PAH (n = 48)	*P*
**Demographics**				
Age, years	57.6 ± 10.4	54.1 ± 9.3	50.1 ± 13.1	0.054
Men, n (%)	12 (46.2)	17 (68.0)	11 (22.9)	0.001
BMI, kg/m^2^	24.1 ± 3.3	26.6 ± 4.3*	23.4 ± 4.5	0.011
Smoking, n (%)	6 (23.1)	6 (24.0)	6 (12.5)	0.363
SBP, mmHg	131.2 ± 16.5	150.3 ± 15.6*	115.9 ± 17.5*	< 0.001
DBP, mmHg	79.2 ± 12.7	96.1 ± 11.5*	68.9 ± 13.4*^†^	< 0.001
MAP, mmHg	98.0 ± 14.5	117.9 ± 12.8*	86.3 ± 15.1*^†^	< 0.001
6MWD, m	–	–	343.4 ± 104.0	–
**Laboratory tests**				
BUN, mg/dL	14.7 ± 4.3	13.7 ± 2.7	17.3 ± 11.9	0.251
Creatinine, mg/dL	0.8 ± 0.2	0.8 ± 0.2	0.9 ± 0.3	0.305
eGFR^‡^, ml/min/1.73m^2^	92.4 ± 19.1	96.7 ± 15.8	82.7 ± 23.2^†^	0.026
NT-proBNP, pg/ml	–	–	1541.1 ± 2530.0	–
**Echocardiography**				
LVEF, %	63.0 ± 7.4	62.7 ± 1.4	67.8 ± 8.2*	0.013
eRVSP, mmHg	27.6 ± 10.8	23.5 ± 9.0	78.1 ± 37.7*^†^	< 0.001
TAPSE, cm	2.5 ± 0.5	2.6 ± 0.3	2.0 ± 0.5*^†^	< 0.001
**Right heart catheterization**				
PAWP, mmHg	–	–	11.0 ± 2.7	–
sPAP, mmHg	–	–	78.2 ± 29.4	–
mPAP, mmHg	–	–	49.9 ± 21.0	–
RAP, mmHg	–	–	12.0 ± 7.1	–
PVR, Wood units	–	–	14.4 ± 8.6	–
**Inflammatory biomarkers**				
TIFA, relative intensity	1.2 ± 0.5	1.9 ± 0.4*	3.25 ± 1.1*^†^	< 0.001
IL-1β, pg/ml	91.6 ± 87.9	154.2 ± 73.5	341.8 ± 110.3*^†^	< 0.001
TNF-α, pg/ml	79.4 ± 51.5	119.9 ± 38.9*	211.2 ± 58.1*^†^	< 0.001

6MWD, 6-min walk distance; BMI, body mass index; BUN, blood urea nitrogen; DBP, diastolic blood pressure; eGFR, estimated glomerular filtration rate; eRVSP, estimated right ventricular systolic pressure; IL-1β, interleukin-1β; LVEF, left ventricular ejection fraction; mPAP, mean pulmonary artery pressure; MAP, mean arterial pressure; NT-proBNP, N-terminal pro-B type natriuretic peptide; PAH, pulmonary arterial hypertension; PAWP, pulmonary artery wedge pressure; PVR, pulmonary vascular resistance; RAP, right atrial pressure; SBP, systolic blood pressure; sPAP, systolic pulmonary artery pressure; TAPSE, tricuspid annular plane systolic excursion; TIFA, tumor necrosis factor-α receptor-associated factor-interacting protein with a forkhead-associated domain; TNF-α, tumor necrosis factor-α.

Post-hoc analysis by Bonferroni test: *P < 0.05, vs. normal; ^†^*P* < 0.05, vs. hypertension.

^‡^eGFR was calculated from the Modification of Diet in Renal Disease equation for Asian.

### Comparisons of TIFA expression and pro-inflammatory cytokines between groups

**Figure 1:**
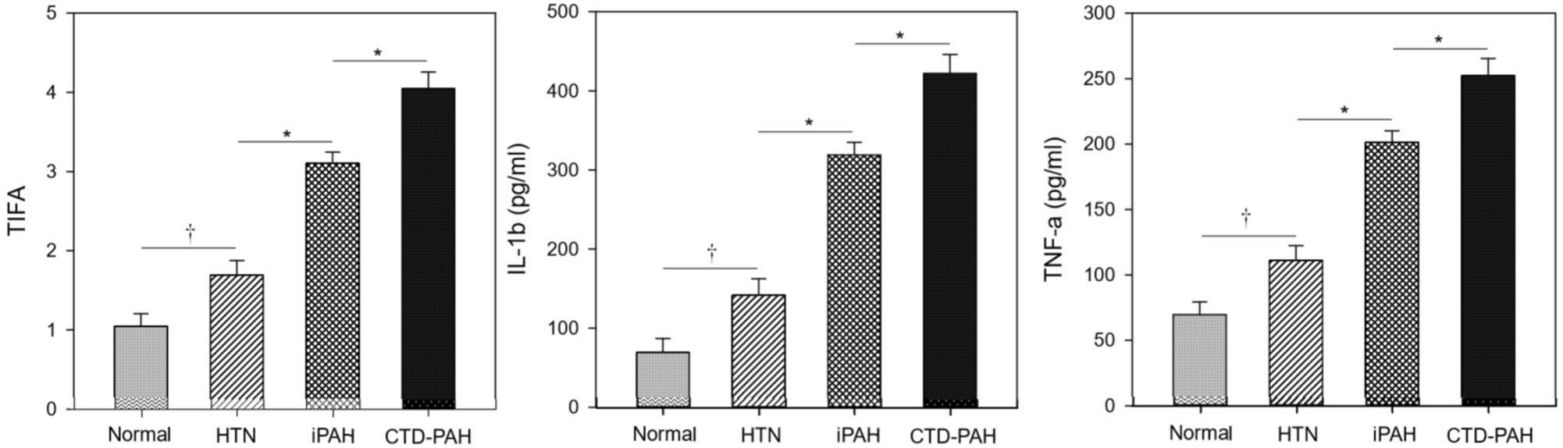
Generalized linear models of TIFA protein expression in PBMCs and plasma levels of IL-1β and TNF-α after adjusting for age, gender, and mean arterial pressure in normal controls, systemic hypertensive (HTN) subjects, patients with idiopathic pulmonary arterial hypertension (iPAH) (n = 32), and patients with connective tissue disease-associated PAH (CTD-PAH) (n = 16). Among all the four groups, patients with CTD-PAH had the highest expression of TIFA protein and plasma levels of IL-1β and TNF-α. Significant between-group differences could be observed in all the three inflammatory biomarkers. *P < 0.001; ^†^*P* < 0.01; All values are expressed as mean ± standard error. IL-1β = interleukin-1β; TIFA = tumor necrosis factor-α receptor-associated factor-interacting protein with a forkhead-associated domain; TNF-α = tumor necrosis factor-α.

Among the study population, PAH patients had significantly higher expression of TIFA protein in PBMCs and plasma IL-1β and TNF-α than the others. In addition, subjects with systemic hypertension also had substantially higher TIFA than the healthy controls (Table 1, and Supplementary Fig. S1). The generalized linear model adjusting for age, gender, and MAP further confirmed the substantial increase in TIFA protein expression and plasma levels of IL-1β and TNF-α in the order of healthy controls, systemic hypertension, idiopathic PAH, and CTD-PAH patients (Fig. 1). It was noteworthy that the statistically significant difference remained between patients with PAH and systemic hypertension.

### Association between TIFA protein expression, pro-inflammatory cytokines, and pulmonary arterial hemodynamics

The associations between TIFA protein expression in PBMCs, baseline characteristics, and invasive pulmonary arterial hemodynamics in patients with PAH are shown in Table 2. TIFA protein expression was positively associated with eRVSP (r = 0.45; *P* < 0.001), IL-1β (r = 0.94; *P* < 0.001), and TNF-α (r = 0.93; *P* < 0.001). In PAH patients with invasive hemodynamics measurements, TIFA positively correlated with sPAP (r = 0.48; *P* = 0.001), mPAP (r = 0.41; *P* = 0.006), and PVR (r = 0.41; *P* = 0.007), while in subjects without PAH, TIFA protein expression was positively associated with MAP (r = 0.449; *P* = 0.002).

**Table 2:** Correlations of TIFA with baseline characteristics and pulmonary arterial hemodynamics by using linear regression analysis.

	r*	*P*
**Total study population (n = 99)**		
Age, years	− 0.137	0.176
BMI, kg/m^2^	− 0.088	0.398
eGFR, ml/min/1.73m^2^	− 0.118	0.299
LVEF, %	0.160	0.139
eRVSP, mmHg	0.450	< 0.001
IL-1β, pg/ml	0.938	< 0.001
TNF-α, pg/ml	0.934	< 0.001
**Subjects with pulmonary arterial hypertension (n = 48)**		
sPAP, mmHg	0.475	0.001
mPAP, mmHg	0.413	0.006
CI, L/min/m^2^	− 0.097	0.547
PVR, Wood unit	0.412	0.007
NT-proBNP, pg/ml	− 0.084	0.575
6MWD, m	− 0.244	0.114
**Subjects without pulmonary arterial hypertension (n = 51)**		
MAP, mmHg	0.449	0.002

6MWD, 6-min walk distance; BMI, body mass index; CI, cardiac index; eGFR, estimated glomerular filtration rate; eRVSP, estimated right ventricular systolic pressure; IL-1β, interleukin-1β; LVEF, left ventricular ejection fraction; MAP, mean arterial pressure; mPAP, mean pulmonary artery pressure; NT-proBNP, N-terminal pro-B type natriuretic peptide; PVR, pulmonary vascular resistance; sPAP, systolic pulmonary artery pressure; TIFA, tumor necrosis factor-α receptor-associated factor-interacting protein with a forkhead-associated domain; TNF-α, tumor necrosis factor-α.

*r: Pearson’s correlation coefficient.

### Diagnostic probability of PAH

**Figure 2:**
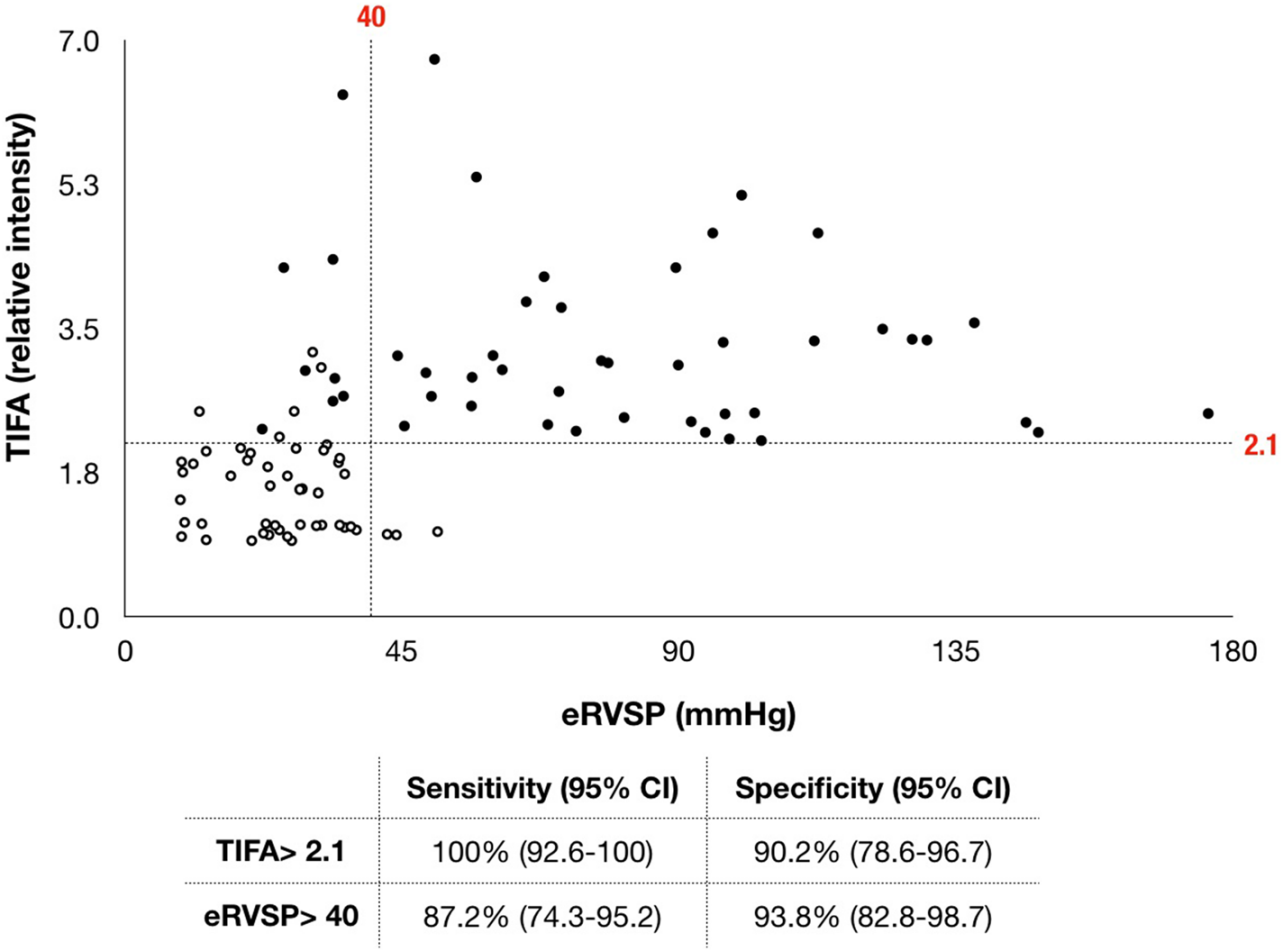
Diagnostic performance of a TIFA value of > 2.1 and an echocardiographic estimation of right ventricular systolic pressure (eRVSP) of > 40 mmHg for pulmonary arterial hypertension (PAH) in the study population. While using TIFA value of 2.1 to predict the PAH diagnosis, the sensitivity was 100% (95% confidence interval, 92.6–100) and the specificity was 90.2% (95% confidence interval, 78.6–96.7). Solid circles = PAH; empty circles = non-PAH. CI = confidence interval.

In unadjusted logistic regression analysis for predicting the presence of PAH, TIFA protein expression in PBMCs, plasma IL-1β, and TNF-α all correlated with the presence of PAH (Table 3). After adjusting for age in the multivariate logistic regression analysis, TIFA, IL-1β, and TNF-α remained as independent predictors of PAH in the study population (Table 3). The ROC curve analysis revealed the area under curve of TIFA to be 0.95 (95% confidence interval: 0.89–0.98) for the prediction of PAH, suggesting that TIFA outperformed eRVSP (0.88, 0.80–0.94). With the use of TIFA (cut-off value, relative ratio of 2.1) to predict PAH diagnosis, the sensitivity was 100% (95% confidence interval, 92.6–100) and the specificity was 90.2% (95% confidence interval, 78.6–96.7) (Fig. 2).

**Table 3:** Logistic regression analysis for the predictors of pulmonary arterial hypertension in the study population (n = 99).

Biomarkers	Unadjusted OR (95% CI) *	*P*	Adjusted OR (95% CI) *^†^	R^2‡^	*P*
**TIFA** (1SD = 1.21)	1.722 (1.349–2.197)	< 0.001	1.722 (1.372–2.163)	0.812	< 0.001
**IL-1β** (1SD = 147.67 pg/ml)	1.488 (1.271–1.742)	< 0.001	1.486 (1.274–1.733)	0.763	< 0.001
**TNF-α** (1SD = 77.71 pg/ml)	1.414 (1.235–1.620)	< 0.001	1.449 (1.255–1.674)	0.763	< 0.001

IL-1β, interleukin-1β; TIFA, tumor necrosis factor-α receptor-associated factor-interacting protein with a forkhead-associated domain; TNF-α, tumor necrosis factor-α.

*Odds ratio (OR) and 95% confidence interval (CI) per-0.1 standard deviation (SD) were presented.

^†^Adjusted for age.

^‡^R^2^ denotes Nagelkerke pseudo R-squared value.

### Causal inference between TIFA protein, pro-inflammatory cytokines, and the presence of PAH

**Figure 3:**
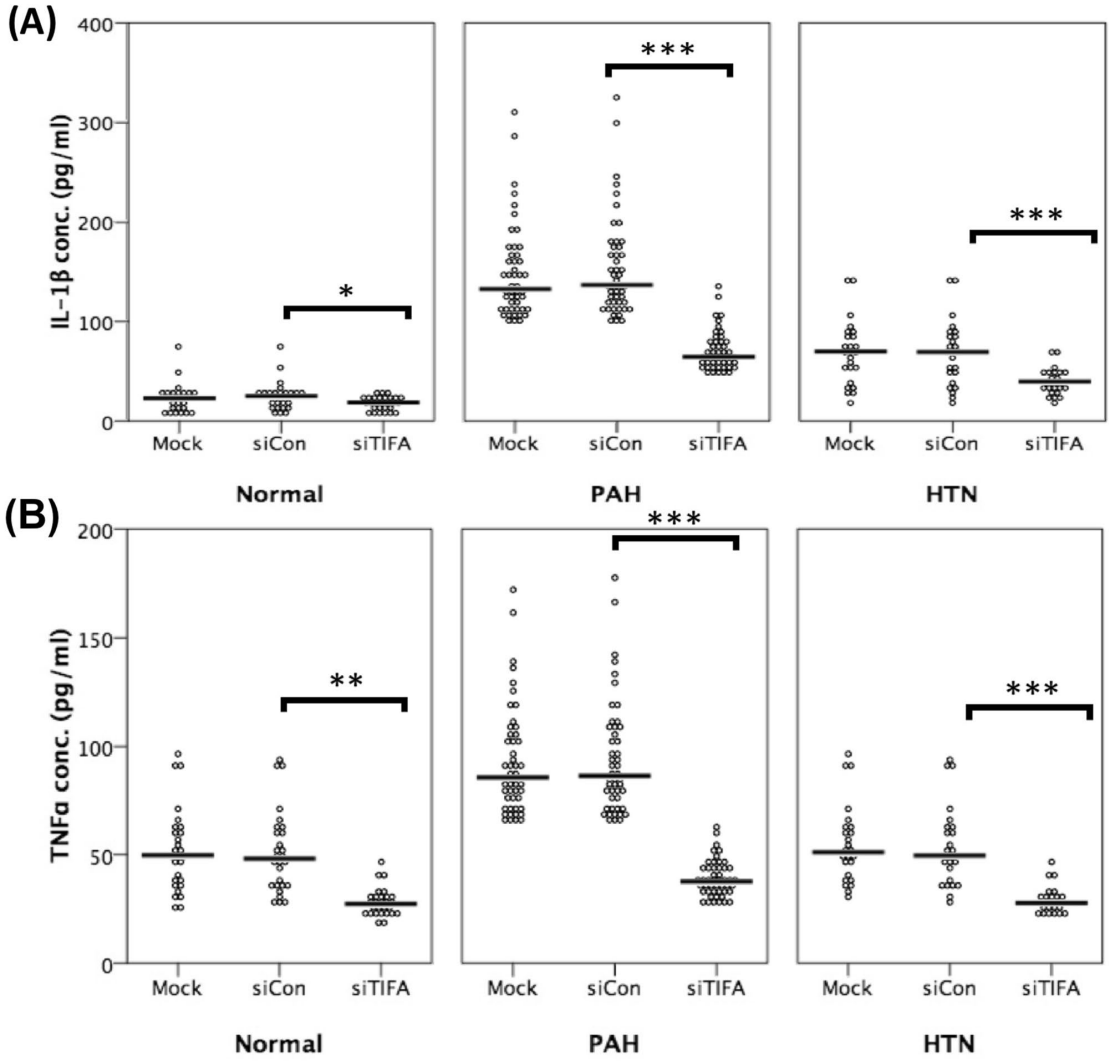
Silencing of TIFA protein expression leads to decreased secretions of (**A**) TNF-α and (**B**) IL-1β in cultured peripheral blood mononuclear cells from patients with pulmonary arterial hypertension (PAH) and systemic hypertension (HTN). *P < 0.05; **P < 0.01; ***P < 0.001. IL-1β = interleukin-1β; siCon = control siRNA; siTIFA = TIFA siRNA; TIFA = tumor necrosis factor-α receptor-associated factor-interacting protein with a forkhead-associated domain; TNF-α = tumor necrosis factor-α.

**Figure 4:**
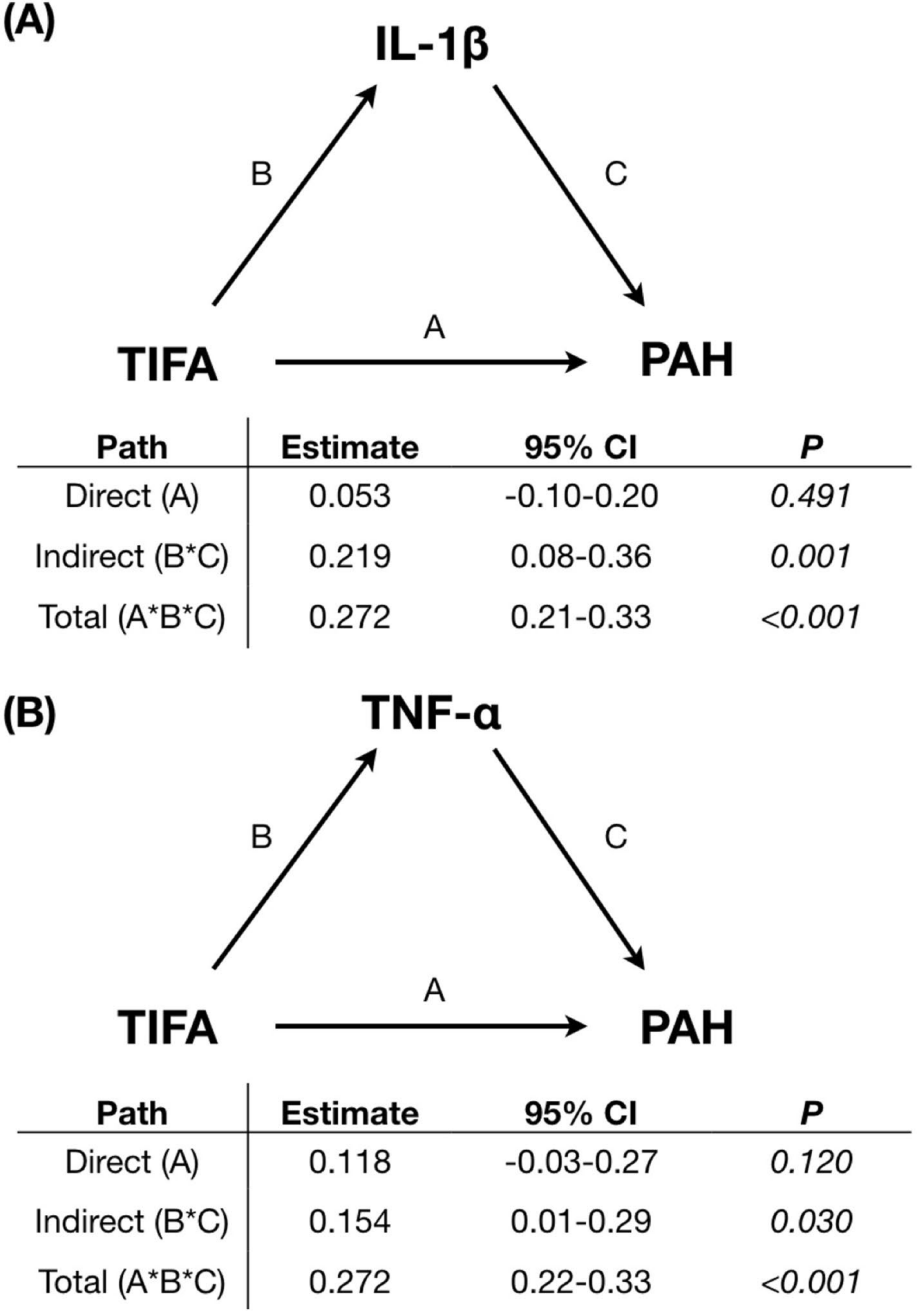
Causal-mediation relationship between TIFA, TNF-α and IL-1β in the pathogenesis of PAH. (**A**) IL-1β as mediator, (**B**) TNF-α as mediator. The effect estimates (hazard ratio) and 95% confidence intervals are reported for all paths (A: direct effect, B*C: indirect effect, A*B*C: total effect). Models were adjusted for age and gender. There were significant indirect and total effects between TIFA and PAH mediated by IL-1β or TNF-α, suggesting that upregulated TIFA protein expression contributes to the development of PAH via the activation of IL-1β (attributable proportion: 80.4%) and TNF-α (attributable proportion: 56.6%).

The ex vivo study showed that siRNA-based TIFA protein expression silencing in PBMCs obtained from systemic hypertension or PAH patients resulted in a significant reduction of both IL-1β and TNF-α levels (Fig. 3A,B). The experimental results can be found as Supplementary Fig. S2 online. To evaluate the potential causal-relationship between TIFA and PAH, we conducted causal-mediation analyses and demonstrated significant indirect and total effects between TIFA and PAH mediated by IL-1β or TNF-α. These results suggest that upregulated TIFA protein expression may contribute to the development of PAH via the activation of IL-1β (attributable proportion [AP], 80.4%) and TNF-α (AP, 56.6%) (Fig. 4).

## Discussion

To the best of our knowledge, this study is the first to demonstrate TIFA protein overexpression in PBMCs of PAH patients and the significant association between TIFA protein expression and plasma levels of IL-1β and TNF-α. TIFA protein expression positively correlated with PVR, an index of PAH severity, and could facilitate PAH diagnosis along with echocardiography. Furthermore, hypertensive subjects had upregulated expression of TIFA, IL-1β, and TNF-α as compared with the healthy controls. These results suggest that subclinical inflammation not only underlies the development of PAH but also systemic hypertension. Ex vivo silencing of TIFA protein expression suppressed the secretion of IL-1β and TNF-α in PBMCs from patients with PAH or systemic hypertension. Causal-mediation analysis further suggested that the effect of TIFA protein overexpression on the development of PAH could be mediated through both IL-1β and TNF-α.

Inflammation is well known to play an important role in PAH pathogenesis. Previous studies revealed the presence of abundant inflammatory cells infiltrating the remodeled pulmonary arterioles and the contribution of elevated circulating cytokines to the underlying inflammation^21,22^. Higher serum levels of inflammatory mediators were shown to correlate with worse hemodynamic indices^10^, and were more predictive of adverse clinical outcomes than the conventional hemodynamic parameters such as CI or mPAP^6,23^. Although the established role of inflammation in PAH has introduced a novel therapeutic paradigm^24^, the detailed inflammatory pathways in PAH remain unclear. Studies have shown that NF-κB is involved in the pathogenesis of PAH^25^. In 12 patients with end-stage idiopathic PAH who underwent lung transplantation, NF-κB was activated in pulmonary inflammatory cells, endothelial cells, and smooth muscle cells^14^. In the present study, we demonstrated the role of TIFA protein, a transducer that sustains the positive feedback signaling in the TNF-α–NF-κB axis^13,19,25^, in the crosstalk between endothelial, smooth muscle, and immune cells following development of cardiovascular diseases in PAH^20,26,27^.

Given that the underlying autoimmune disease may confound TIFA protein expression level, we further stratified PAH patients based on their etiologies into idiopathic PAH and CTD-PAH. As expected, the levels of inflammatory factors were higher in the CTD-PAH subgroup and may potentially indicate worse outcomes than idiopathic PAH^28^. The differences in TIFA protein expression observed in our study indicate a spectrum of varying degrees of inflammation in different etiologies of PAH. The significantly higher expression of TIFA protein in idiopathic PAH also manifested the role of inflammation even in the absence of CTD.

Aside from the PAH group, we also observed higher expression of inflammatory biomarkers in hypertensive subjects than in healthy controls. Mirhafez et al*.* demonstrated IL-1α, interferon-γ, and IL-10 as independent predictors of higher SBP and presence of systemic hypertension in a cross-sectional study involving 155 hypertensive patients and 148 healthy subjects^29^. In a meta-analysis of 142,640 participants, Jayedi et al*.* suggested circulating C-reactive protein (CRP) and IL-6 to be associated with the risk of developing systemic hypertension^30^. Although the exact mechanism remains to be elucidated, evidence shows that oxidized lipids may trigger the formation of atherosclerotic plaques and induce cytokine production via NF-κB activation^31^. By altering endothelial function, chronically activated inflammation appears to increase arterial stiffness and contribute to the development of systemic hypertension^32,33^. In line with the findings, our study shows that hypertensive subjects had significant upregulation of TIFA protein expression in PBMCs as well as higher plasma IL-1β and TNF-α levels. The results not only support the role of inflammation in systemic hypertension but also point out TIFA protein as a sensitive biomarker, adjunctive to other inflammatory mediators, to surrogate the extent of vascular remodeling underlying systemic hypertension.

The present study has several limitations. First, we reported a significant association between TIFA and PAH from a cross-sectional evaluation in a relatively small sample-sized study. Larger-scale studies with longitudinal follow-up are warranted to investigate the change in TIFA under PAH-specific treatment and to determine if it is a reliable marker to reflect PAH disease activity. Second, most patients from PAH group were women, which was compatible with the disease prevalence. Although gender was adjusted in the multivariable analysis, gender-specific variation should be considered, especially in the inflammatory signaling pathways. Third, although the present study shows that TIFA is involved in the pathogenesis of PAH, TIFA may not be specific to PAH and could also be elevated in the presence of underlying autoimmune diseases. Future studies should gather data on the associations between TIFA and other inflammatory markers, such as CRP level, and determine whether CTD-PAH patients have higher expression of TIFA protein than CTD patients without PAH.

TIFA protein expression in PBMCs is markedly increased in patients with PAH and positively correlates with PAH severity. As TIFA acts as an imperative transducer that propagates inflammatory responses by activating NF-κB signaling pathways^13,19,20,34^, the study demonstrates the association between the upstream TIFA protein and the downstream plasma IL-1β and TNF-α in PAH. The findings of the present in vitro study may be considered as a promising aspect for further investigation in common animal models of PAH. In addition, the increased expression of TIFA protein in hypertensive subjects also indicates TIFA as a potentially sensitive marker of subclinical inflammation underlying the pathogenesis of PAH as well as systemic hypertension. It is worth exploring the mechanisms of how TIFA initiates the progression of PAH and systemic hypertension and the feasibility of TIFA as a novel therapeutic target in future studies.

## Supplementary Information

Supplementary Information.

## Data Availability

The data supporting the findings of this study are available from the corresponding author upon reasonable request.
