# Supplementary material for: TIFA protein expression is associated with pulmonary arterial hypertension
Source: Sci Rep. 2021 Jul 8;11:14140. doi: 10.1038/s41598-021-93582-1 (PMC8266829; doi:10.1038/s41598-021-93582-1)

**TIFA Protein Expression is Associated with Pulmonary Arterial Hypertension**

**Short title: *TIFA protein expression in PAH***

Hao-Chih Chang, M.D.^a,c#^; Tong-You Wade Wei, Ph.D.^e#^; Pei-Yu Wu, Ph.D.^e^; Ming-Daw Tsai, Ph.D.^e^; Wen-Chung Yu, M.D.^a,b,c^; Chen-Huan Chen, M.D.^b,c,d^; Shih-Hsien Sung*, M.D., Ph.D.^a,b,c,d^

^a^ Division of Cardiology, Department of Medicine, Taipei Veterans General Hospital, Taipei, Taiwan

^b^ Cardiovascular Research Center, National Yang Ming Chiao Tung University, Taipei, Taiwan

^c^ Department of Internal Medicine, National Yang Ming Chiao Tung University College of Medicine, Taipei, Taiwan

^d^ Institute of Public Health, National Yang Ming Chiao Tung University College of Medicine, Taipei, Taiwan

^e^ Institute of Biological Chemistry, Academia Sinica, Taipei, Taiwan

^#^ Equal contribution

*** Corresponding author information:**

Shih-Hsien Sung, M.D., Ph.D.

Address: No. 201, Sec. 2, Shipai Road, Beitou District, Taipei, Taiwan

Division of Cardiology, Department of Medicine, Taipei Veterans General Hospital, Taipei, Taiwan

Tel: +886-2-2875-3873

Fax: +886-2-2877-1746

E-mail: [mr.sungsh@gmail.com](mailto:mr.sungsh@gmail.com)

**Supplementary Figure S1. Overexpression of TIFA protein in patients with PAH.**


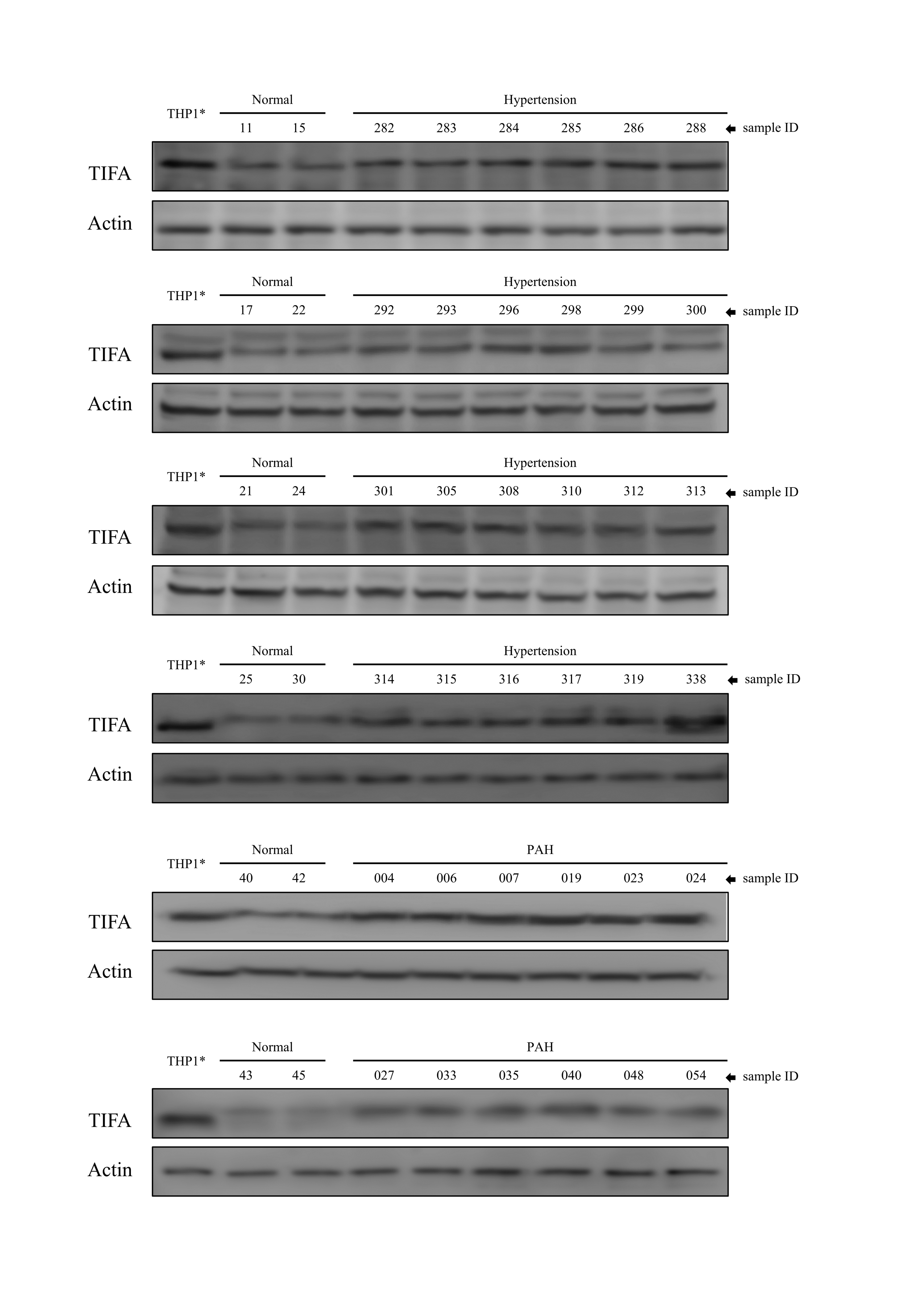


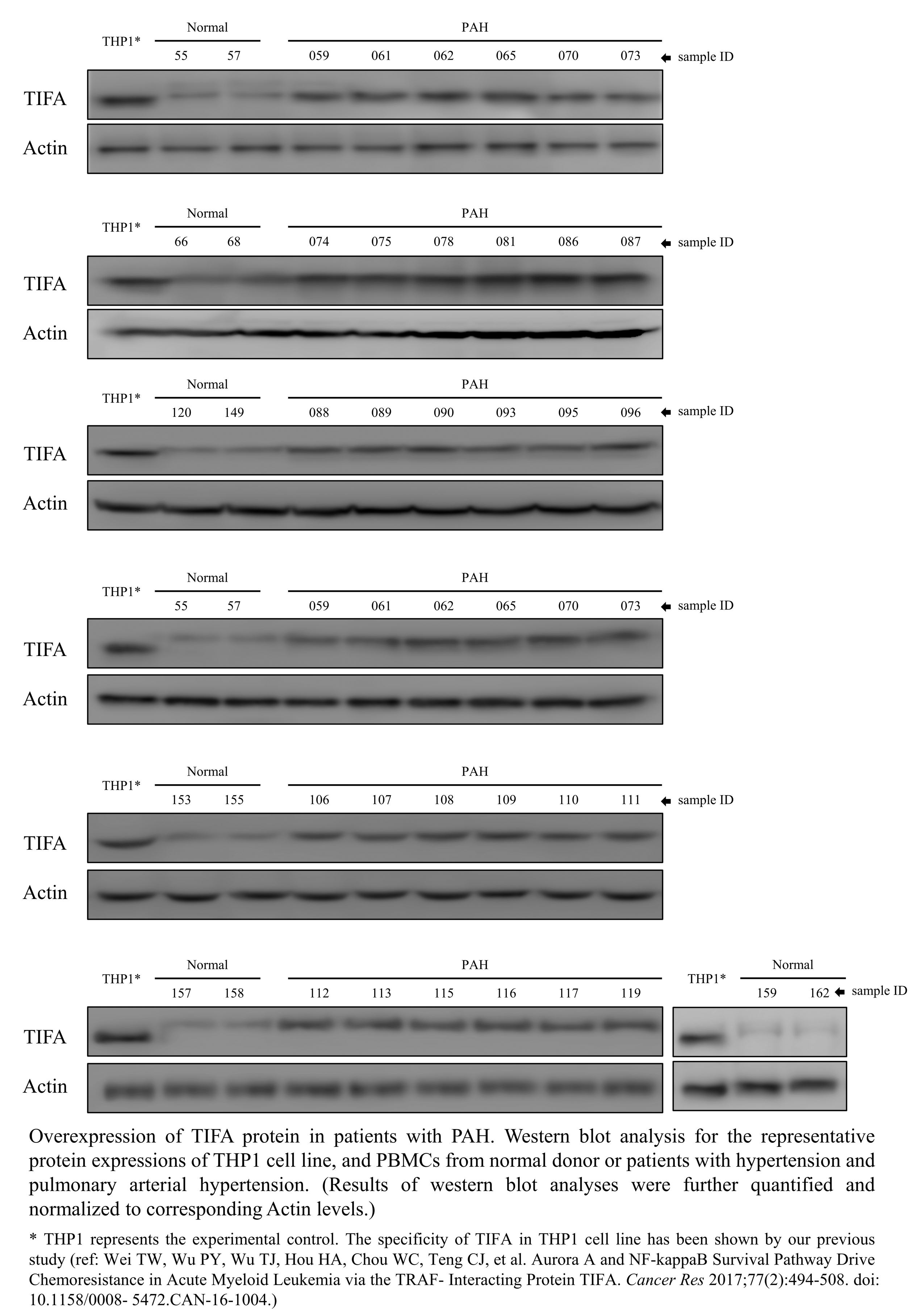


Western blot analysis for the representative protein expressions of THP1 cell line, and PBMCs from normal donors or patients with systemic hypertension and pulmonary arterial hypertension. (Results of western blot analyses were further quantified and normalized to corresponding Actin levels.)

* THP1 represents the experimental control. The specificity of TIFA in THP1 cell line has been shown by our previous study.[1]

**Supplementary Figure S2. siRNA-induced TIFA silencing in THP1 and PAH PBMCs.**


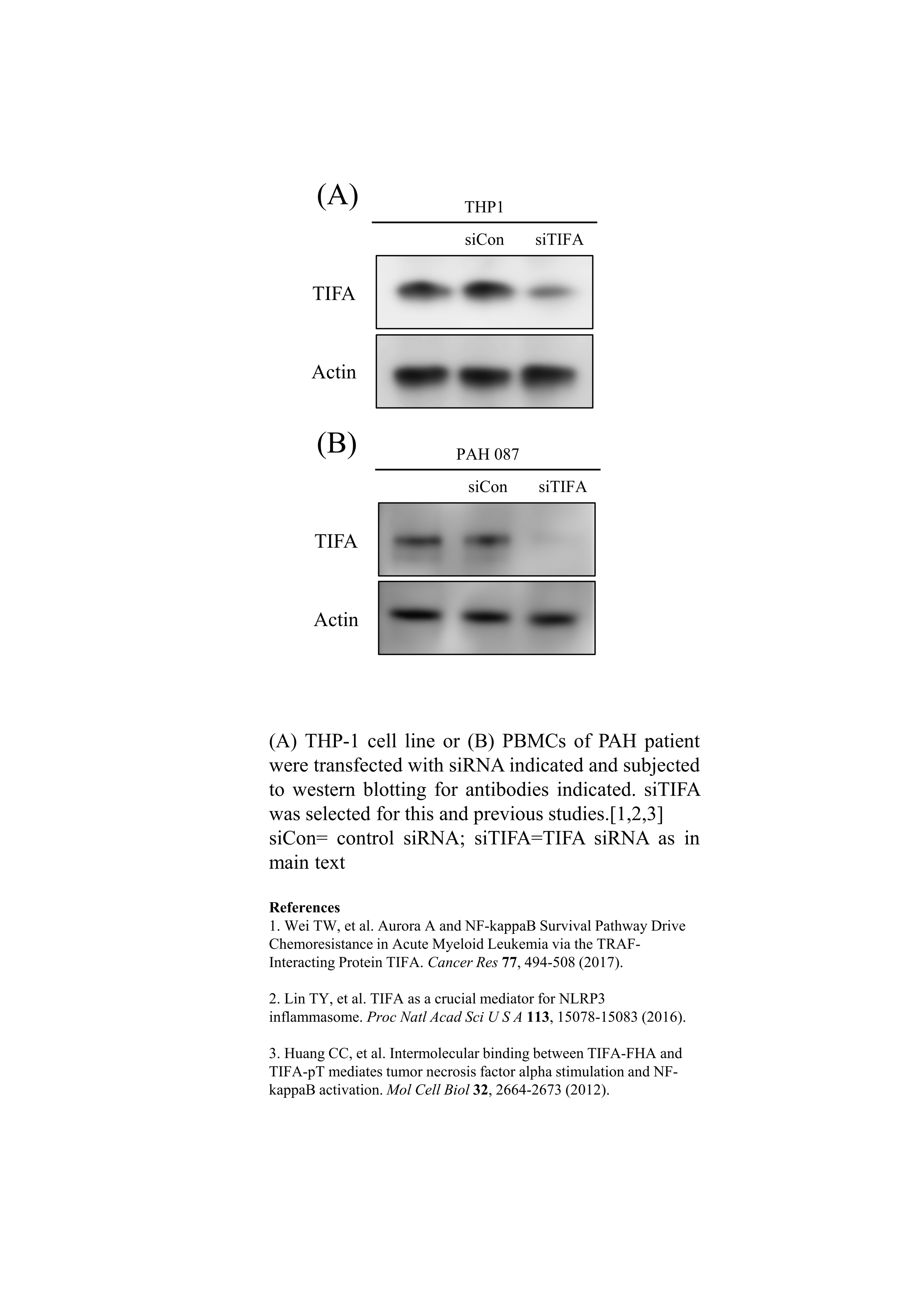


(A) THP-1 cell line or (B) PBMCs of PAH patient were transfected with control or TIFA siRNA and subjected to western blotting with indicated antibodies. siTIFA was selected for this and previous studies.[1,2,3]

siCon= control siRNA; siTIFA=TIFA siRNA as in main text

**References**

1. Wei TW, et al. Aurora A and NF-kappaB survival pathway drive chemoresistance in acute myeloid leukemia via the TRAF-interacting protein TIFA. *Cancer Res* **77**, 494-508 (2017).
2. Lin TY, et al. TIFA as a crucial mediator for NLRP3 inflammasome. *Proc Natl Acad Sci U S A* **113**, 15078-15083 (2016).
3. Huang CC, et al. Intermolecular binding between TIFA-FHA and TIFA-pT mediates tumor necrosis factor alpha stimulation and NF-kappaB activation. *Mol Cell Biol* **32**, 2664-2673 (2012).

**Full-length gels/blots**

Supplementary Figure S1. Overexpression of TIFA protein in patients with PAH.


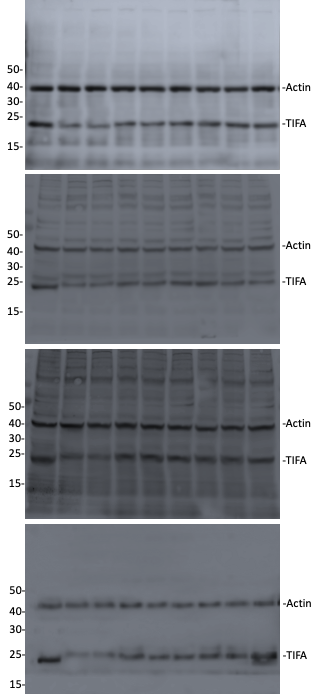


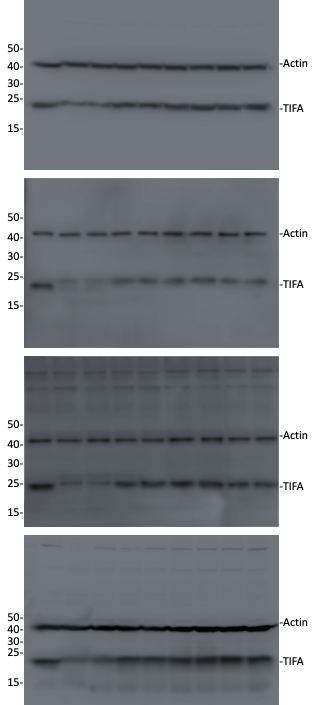


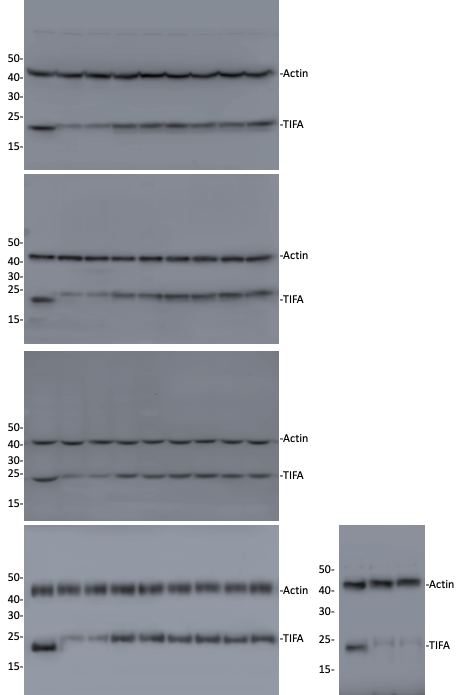


Supplementary Figure S2. siRNA-induced TIFA silencing in THP1 and PAH PBMCs.


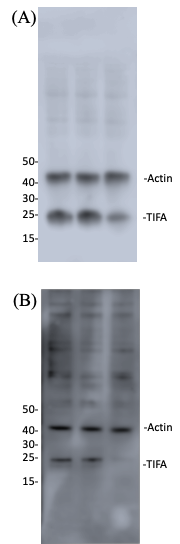

Supplement: Supplementary file 1 — Supplementary Information. [file 41598_2021_93582_MOESM1_ESM.docx]
